# Patient-Reported Outcomes in a Nationally Representative Sample of Older Internet Users: Cross-sectional Survey

**DOI:** 10.2196/16006

**Published:** 2021-11-24

**Authors:** Gul Seckin, Susan Hughes

**Affiliations:** 1 Department of Sociology University of North Texas Denton, TX United States; 2 Department of Sociology Ouachita Baptist University Arkadelphia, AR United States

**Keywords:** internet, information, health, communication, strain, education, eHealth literacy

## Abstract

**Background:**

The rapid diffusion of the internet has decreased consumer reliance on health care providers for health information and facilitated the patients’ ability to be an agent in control of their own health. However, empirical evidence is limited regarding the effects of health-related internet use among older adults, which is complicated by the proliferation of online health and medical sources of questionable scientific accuracy.

**Objective:**

We explore the effects of health-related internet use, education, and eHealth literacy on medical encounters and patient-reported outcomes. Patient-reported outcomes are categorized into two dimensions: (1) self-reported health problem and (2) affective distress (feeling worried and anxious) due to information obtained. We were particularly interested in whether education and eHealth literacy moderate the association between perceived strain in medical encounters and patient-reported outcomes.

**Methods:**

Our study sample consisted of online panel members who have used the internet as a resource for health information, randomly drawn from one of the largest probability-based online research panels. This paper specifically reports results obtained from older panel members (age≥60 years: n=194). First, we examined descriptive statistics and bivariate associations (Pearson correlations and independent samples *t* tests). We used hierarchical ordinary least squares regression analyses by running separate regressions for each patient-reported outcome. In model 1, we entered the main effects. In model 2, technology and medical encounter variables were included. Model 3 added the statistical interaction terms.

**Results:**

Age (*β*=–.17; *P*=.02), gender (*β*=–.22; *P*=.01), and medical satisfaction (*β*=–.28; *P*=.01) were significant predictors of self-reported health problems. Affective distress was positively predicted by gender (*β*=.13; *P*=.05) and satisfaction with medical encounters (*β*=.34; *P*<.001) but negatively predicted by education (*β*=–.18; *P*=.03) and eHealth literacy (*β*=–.32; *P*=.01). The association between experiencing a health problem in relation to health-related internet use and perception of strained medical encounters was greater among respondents with lower levels of education (*β*=–.55; *P*=.04). There was also a significant interaction between education and eHealth literacy in predicting the level of affective distress (*β*=–.60; *P*=.05), which indicated that higher levels of education predicted lower averages of feeling anxiety and worry despite lower eHealth literacy. Older women reported higher averages of affective distress (*β*=.13; *P*=.05), while older men reported higher averages of experiencing a self-reported health problem (*β*=–.22; *P*=.01).

**Conclusions:**

This study provides evidence for the effect of health-related internet use on patient-reported outcomes with implications for medical encounters. The results could be used to guide educational and eHealth literacy interventions for older individuals.

## Introduction

### Internet in the Health Care Landscape

Rapid diffusion of information and communication technologies along with the self-care/self-help movement has increased the use of the internet for health information while decreasing consumer reliance on health care providers. Health professionals increasingly interact with health care consumers who want to relinquish their dependent role [1]. Most individuals consult the internet to find information for at least one health topic before visiting their health care provider, making it one of the most common online activities [2-6]. Historically, health care providers used to be the information source for their patients, which ensured patient acceptance of the health care provider’s informational authority and their compliance with the treatment plan decided for them. The internet has transformed the landscape of health communication and information. As the use of the internet as a source of information has substantially increased, a more participatory model of care has increasingly become prominent in US medical care, which has led to changes in the structure of the traditional paternalistic health care paradigm [6,7]. In the age of expanding digital information technologies, the internet, as an important source of health information, has transformed the ways that consumers use health information, interact with their physicians, and receive health care services [8].

### Health-Related Internet Use and Older Adults

Health care providers increasingly interact with older adults who gather health information from the internet. Although older adults are more likely to prefer health care providers as their trusted source of health information, the internet presents convenient options to obtain health and medical information, making it one of the major reasons for internet use [9-14]. In fact, aging adults in the United States represent the fastest growing group of internet users who view online information as a resource to support their health and well-being [9-11,15,16]. The Pew Research Center reports that almost 70% of computer-connected seniors use the internet [17]. The societal focus on successful aging strategies and increased quality of life in later life has provided an impetus to empower older adults as health care consumers. Health information gathering is among the major motivations for using the internet among aging baby boomers who have more experience with information and communication technologies compared to previous cohorts of older adults [3,9,11,18]. Technology acts as a buffer against health challenges in later life by increasing access to informational resources that allows older adults to be proactive in shaping their health outcomes [9-14,17,19]. With these developments, more research attention is focusing on how digital health information influences doctor-patient interactions and health outcomes among older internet users [6,8,9,20-23].

### Concerns for Information Quality and Medical Encounters

The internet presents new multiple options for older adults to gather information to support their health care [10,24,25]. eHealth technologies associated with health management, promotion, and disease prevention continue to grow with new smartphone and iPad apps, mobile health tools, and social media. Although there is a generally favorable perspective toward use of the internet to acquire health information [21], the ease of access to inaccurate information on the web or the misinterpretation of the information poses potential risks to health and well-being. Despite the fact that aging baby boomers are better educated and more technology savvy than previous cohorts, inaccurate information on the internet represents a major challenge to an informed use of information technology among older adults [9,11,13,26-31]. Older adults are of particular concern as they are likely to have lower eHealth literacy than younger adults despite their increased needs for health information [26]. Research reports that just over 10% of US adults have adequate eHealth literacy [16]. This percentage is only about 3% among older adults [32-40]. The presence of questionable information sources on the internet, which ranges from personal blogs to non–peer-reviewed medical advice and commercial websites, hinders the proactive and informed use of the internet for health information [41]. Despite these challenges, older adults’ use of the internet for health purposes and their ability to evaluate online information and, consequently, possible negative health outcomes remain an understudied area of research that is further complicated by the rapid proliferation of web sources of questionable scientific accuracy and trustworthiness [9,11,13,23-28,30,31].

Prior research has shown that individuals with higher levels of education and eHealth literacy levels are better able to engage in an assessment of information quality and to deploy the information appropriate in the management of their health [3,6,20,22,25,28,33,34,42-45]. Even though the internet can be an efficient tool to inform oneself, users’ limited skills to make quality and credibility assessments of online health information limits health care providers’ endorsement of the internet as a beneficial informational resource for their patients [46,47]. Although concerns about most patients’ inability to appraise online health information and access inaccurate information due to their limited eHealth literacy have been noted, researchers have mostly focused on positive effects of internet use on the relationship between patients and their physicians [41].

Internet use for health-related information is also associated with challenges in the doctor-patient relationship, when a health consumer believes that online information is as good as information provided by their provider [43,48-50]. Researchers found that trust in information sources affect patients’ attitudes and behaviors, and their satisfaction with interactional and communicational aspects of the clinical encounters [51]. Medical directives may come from providers, but health consumers’ choices are influenced by a wide range of alternative sources of information on the internet [24,48,52,53]. Information retrieval from noncredible internet sources may particularly hinder a patients’ ability to form effective collaboration with their health care provider [11,16,21,54-56], which increases the importance of eHealth literacy skills. Insufficient eHealth literacy particularly presents challenges for older health consumers in an increasingly digitized society that places primary responsibility on individuals for their health care, a phenomenon to which some scholars have referred to as a “perfect storm” [57].

Distrust in the doctor’s opinion, diagnosis, or treatment and subsequently nonadherence with the treatment plan may occur when patients find information that is not aligned with the doctor’s approach [6,32,49,50,57-59]. Furthermore, a health care provider may also feel that the patient does not trust their knowledge and expertise or may feel that the internet information is being used to test the health care provider’s knowledge [1,6,23,35,36,57]. Chung [7] found that patients who experienced poor health perceived health providers’ reactions to their use of online information to be negative. About 40% of physicians think that internet use may harm the quality of the physician-patient relationship, given the vast amount and varying quality of information [49].

Health-related internet use might also be a source of frustration as “online information can add a new interpretive role to physicians’ responsibilities during consultations” [6], increasing the amount of time and labor with misinformed patients, particularly if they have more questions or request additional treatments or medications [1,6,7,23,32,47,49,60,61]. If a health care provider dismisses information, a patient may feel frustrated and concerned that their use of the internet poses a barrier to achieving satisfactory doctor-patient interactions [7,24,36,49,59]. Consequently, internet use for health information may have an effect on medical encounters that is not always for the better [1,6,23,32,36,49,54,56,62,63]. Thus, there has been growing recognition that eHealth literacy should be taken into account to achieve an effective doctor-patient communication and health care partnership [11].

### Theoretical Approaches

The Transaction Model of eHealth Literacy (TMeHL) informs the theoretical approach of our paper [27]. The transactional aspects of eHealth literacy refers to the communicative skills of an online information user in exchanging information with medical professionals [27]. TMeHL posits that interpersonal dynamics in social contexts drive the transactional process of communication [3]. People who possess eHealth literacy are likely to develop competences and skills, which improve their ability to communicate with their physicians, such as the ability to ask informed questions and better understand new information, which in turn are likely to result in more satisfaction in patient interactions with physicians [3,30,41]. Therefore, effective health information exchange is dependent on the interpersonal dynamics between the patient and provider. Individuals with higher eHealth literacy are better able to make appropriate assessments of information quality and credibility, and to deploy this information as a resource in the management of their health [27,28]. Accordingly, this perspective considers education and eHealth literacy to be essential intrapersonal resources to engage with online health information effectively that would contribute to the quality of health care interactions.

A related theoretical framework of the paper is the Transactional Model of Communication (TMC). The TMC posits that the interactions among communicators may include varying levels of noise that can interfere with the process of communication [27]. Importantly, noise may hinder a patient’s ability to appropriately consume and apply eHealth information or participate in successful exchanges of information with health providers particularly for those who have low levels of eHealth literacy [27]. The factors that induce *noise* within the context of medical encounters can include use of various questionable sources of information on the internet, ranging from personal blogs to non–peer-reviewed medical advice on commercial websites.

Theoretical underpinnings of the TMeHL and TMC suggest that eHealth literacy promotes a positive eHealth experience when interacting with medical professionals. eHealth literacy assists internet users to sort through online health-related information that may result in improved interactions with health care professionals [27,30]. eHealth literacy may also negate the detrimental effects produced from noise in eHealth contextual factors (eg, health and medical information of questionable accuracy on the internet) [27]. Seçkin et al [30] also identified communication with health providers as a core component of eHealth literacy, a dimension they refer to as interactional literacy. A consumer of online health information resources must possess an eHealth literacy skill set to support positive eHealth experiences and patient-provider interactions while reducing noise that may impact the transaction [27]. Paige et al [27] also pointed to a need for research to explore how eHealth literacy may serve as a moderator to buffer the negative effects of personal or relational impediments or barriers in medical encounters that limit the effective use of information technology in the management of patient health. Thus, research increasingly points to the need to examine the moderating role of the eHealth literacy on patient-physician dynamics, including interpersonal tensions and strain that might stem from consuming either too much or irrelevant information or locating erroneous information from noncredible websites [3,27,41]. Although research over the last decade has examined technological or personal barriers that impact eHealth literacy, this research has delivered limited understanding of the communicational and transactional processes, which are highly salient to a positive patient experience during medical encounters [27]. Research on eHealth literacy is infrequently framed in a way that demonstrates its transactional nature, which continues to limit our full comprehension of eHealth literacy in the digital age [27]. Moreover, in contrast to the rapidly growing literature focused on positive aspects of using online health information, little research has examined adverse outcomes of health-related internet use [21,61,63].

### Research Goals and Objectives

The previously discussed issues led us to examine the effects of education and eHealth literacy separately as independent predictors and as joint moderators in our paper, which captures the transactional nature of eHealth literacy within the context of medical encounters for older individuals’ subjectively reported health-related outcomes. This paper seeks to connect eHealth literacy with interactional dynamics of medical encounters that affects patients’ experience of medical encounters and subjective health outcomes.

eHealth literacy encompasses both patient information appraisal behavior (behavioral eHealth literacy) and communicational skills used by the patient when interacting with their health care providers (interactional eHealth literacy), which supports a successful acquisition of health information and meaningful patient-physician interactions [27,30]. Prior research shows that internet users with less education tend to have lower scores on health literacy measures, a trend that adversely affects satisfaction with doctor-patient interactions [64]. Seckin et al [30] also reported significant differences in eHealth literacy among internet users based on their educational attainment. Building on previous research, we suggest that education and eHealth literacy are intrapersonal resources that facilitate the exchange of information between self-informed patients and health care professionals [27]. One of the contributions of this paper to the literature lies in its ability to capture the transactional importance of eHealth literacy, which is important for fostering collaboration between a health care provider and patient-consumer.

We specifically examined whether eHealth literacy predicts patient-reported negative outcomes, whether education moderates the association between eHealth literacy and negative outcomes, and whether both eHealth literacy and education moderate the association between the perception of strain in the health care provider-patient relationship and negative patient-reported outcomes. It is important to understand these relationships because the consequences for using low-quality, misleading, or false information could endanger health [19]. To our knowledge, no prior research has examined whether education and eHealth literacy moderate the effect of perceived strain in medical encounters on patient-reported outcomes among older internet users [58].

## Methods

### Sample

Respondents were randomly sampled from the online probability-based research panel developed by Knowledge Networks (KN). KN used an address-based sampling frame derived from the US Postal Service Delivery Sequence File, which covers 97% of US households, thereby maximizing sample representativeness. Analyses are representative of the larger US population because all KN panel households were selected randomly with a known probability of selection, and our study respondents were further randomly selected from the larger panel. KN sent a recruitment email invitation to 1315 randomly selected panel members who were asked whether they sought health-related information on the internet. We obtained a 66% (n=870) response rate Of those who responded to the recruitment email, 710 cases qualified for the study by confirming their use of the internet to the screening question and completed the online survey. This paper specifically focuses on the internet users who were 60 years or older (n=194).

### Measures

Patient-reported outcomes included the extent to which study participants have ever experienced a health problem (self-reported health problem) as a result of using the internet information and felt worried or anxious (affective distress) because of gathering health or medical information from the internet. Responses ranged from 1 (strongly disagree) to 5 (strongly agree). We examined these patient-reported outcomes individually by performing item-based analyses.

Health-related internet use was measured with eight items (Multimedia Appendix 1) such as whether respondents “seek information on the internet to self-diagnose” and whether they “use information from the internet to make treatment decisions.” Response options ranged from 1 (never) to 5 (always). An index score was created by computing the average score of the eight items (full sample: mean 1.86, SD 0.63; older adult subsample: mean 1.79, SD 0.65). The Cronbach alpha reliability coefficient for the composite scale is .83 in the full sample and .90 in the older adults subsample.

Patient nonadherence was measured by whether respondents “doubt diagnosis or treatment of a health care provider if it conflicts with information on the internet,” “change their willingness to accept a health care provider’s treatment after reading information on the internet,” and “change a health care provider’s treatment after reading information on the internet.” Response options ranged from 1 (never) to 5 (always). An index score was created by computing the average score of the three items (full sample: mean 4.67, SD 1.72; older adult subsample: mean 1.71, SD 0.63). The Cronbach alpha reliability coefficient for the composite scale is .71 in the full sample and .73 in the older adults subsample.

Satisfaction with health care provider-patient relationship (referred to as medical satisfaction in tables) was assessed by asking respondents to indicate the extent of their agreement with statements such as “information on the internet helps me to communicate more effectively with health providers during appointments” and “I receive more information from health providers as a result of gathering information from the internet.” Responses ranged from 1 (strongly disagree) to 5 (strongly agree). An index score was created by computing the average score of the six items (full sample: mean 3.16, SD 0.61; older adult subsample: mean 3.17, SD 0.57). The Cronbach alpha reliability coefficient for the composite scale is .86 in the full sample and .91 in the older adults subsample. A complete list of items is provided in Multimedia Appendix 1.

Respondents were also asked a single item about perceived strain in health care provider and patient relationship with the statement “interactions with health providers have become strained as a result of bringing in health or medical information from the internet to my appointments” (1, strongly disagree, to 5, strongly agree). Item-based analyses examined whether differential patterns of associations were obtained for perceived strain on this item instead of reverse coding it and including in the composite scale for medical satisfaction, which ensured detailed results were obtained for dissatisfaction with medical encounters.

eHealth literacy was measured with the 19-item eHealth Literacy Scale (e-HLS) instrument [30], as this instrument reflects skills associated with evaluating, communicating, and using information to make informed decisions when it comes to health care such as whether respondents check for credentials and institutional affiliations of those who provide information on websites (Multimedia Appendix 1). Responses ranged from 1 (never) to 5 (always). An index score was created by computing the average score of the 19 items (full sample: mean 2.51, SD 0.77; older adult subsample: mean 2.53, SD 0.81). The Cronbach alpha reliability coefficient for the composite scale is .93 in the full sample and the older adults subsample. The responses to the e-HLS items were recoded into two groups for independent samples *t* test analyses to represent low health literacy and average to high health literacy. Respondents who indicated 1 (never) and 2 (rarely) on a five-point Likert scale for each item on the e-HLS instrument were coded as the low eHealth literacy group using SPSS (IBM Corp) procedures for recoding data. Respondents who indicated sometimes to always (3=sometimes, 4=often, and 5=always) on e-HLS items were coded as the average to high eHealth literacy group.

Sociodemographic covariates included the following: age was measured as a continuous variable; sex was coded as male (0) and female (1); race/ethnicity was coded as Caucasian (0) and minority (1); education was coded as high school or less (1), some college or associate degree (2), college degree (3), and postgraduate degree (4); income was collapsed into four groups: US $29,999 or less (1); US $30,000-$59,999 (2); US $60,000-$99,999 (3); and US $100,000 and above (4); marital status was coded as married (0) and unmarried (1).

### Statistical Analysis

First, descriptive and bivariate analyses (correlational analyses and *t* tests) were performed. Regression diagnostics were conducted on the residuals to make sure the underlying assumptions of multiple regression analysis (ie, homoscedasticity) were met. Hierarchical ordinary least squares regression models examined the associations among variables and their relative predictive strengths. Model 1 in each table represents the main effects for sociodemographic characteristics. Model 2 was adjusted for technology and medical encounter variables. The interaction terms (eHealth literacy × strain, eHealth literacy × education, and education × strain) were entered in the final step (model 3). This analytical approach allowed examination of the changes in the relative effect of each covariate on the outcome variables. Parallel regression models for each patient-reported outcome were performed.

## Results

The complete study sample included respondents aged 18-93 years (mean 48.8, SD 16.4). Respondents 60 years and older (the focus of this paper) represented about 27% (194/710) of the total sample (mean 68.7, SD 7.4). About 40% (73/194, 37.6%) of the older respondents had a college degree or higher, and just over half of the respondents (99/194, 51.1%) reported an income level of US $60,000 or more. Women accounted for more than half of the sample (107/194, 55.2%). About 60% were married (121/194, 62.4%), and just over 80% (160/194, 82.5%) were Caucasian. Descriptive statistics of the study variables in the older sample of health-related internet users is provided in Table 1.

As shown in Table 2, we also examined independent samples *t* tests to investigate the effect of eHealth literacy levels on study covariates. Older adults with higher levels of eHealth literacy reported lower averages for perceived strain in medical encounters (*t*_194_=2.92; *P*=.01). They also reported lower averages for affective distress (*t*_194_=2.11; *P*=.04) and more satisfaction with medical encounters (*t*_194_=4.70; *P*<.001). There are also significant differences in the averages for nonadherence (*t*_194_=5.06; *P*<.001) and self-reported health problems in relation to internet use (*t*_194_=1.93; *P*=.05).

Correlational analyses indicated that education is positively associated with eHealth literacy (*r=*0.27; *P*<.001) but negatively associated with strain in medical encounters (*r*=0.16; *P*=.03). eHealth literacy has a positive association with satisfaction with medical encounters (*r*=0.40; *P*<.001) but a negative association with perceived strain (*r*=–0.18; *P*=.01). We also found that affective distress is negatively related to education (*r*=–0.21; *P*=.01) and eHealth literacy (*r*=–0.16; *P*=.03) but positively related to health-related internet use (*r*=0.17; *P*=.02) and strained medical encounters (*r*=0.17; *P*=.01). Experiencing a self-reported health problem is positively associated with health-related internet use (*r*=0.16; *P*=.02) and nonadherence (*r*=0.17; *P*=.04).

**Table 1:** Descriptive statistics (N=194).

Research variables	Participants, mean (SD)
Health-related internet use (range 1-5)	1.79 (0.65)
eHealth literacy (range 1-5)	2.53 (0.81)
Medical satisfaction (range 1-5)	3.17 (0.57)
Perceived strain (range 1-5)	2.40 (0.77)
Nonadherence (range 1-5)	1.71 (0.63)
Self-reported health problem (range 1-5)	1.04 (0.26)
Affective distress (range 1-5)	2.34 (0.80)

**Table 2:** Covariates stratified by eHealth literacy level (N=194).

Covariates	Low eHealth literacy, mean (SD)	Average to high eHealth literacy, mean (SD)	*T* test (*df*)	*P* value
Medical satisfaction	3.09 (0.58)	3.51 (0.66)	4.70 (194)	.001
Perceived strain	1.56 (0.50)	1.36 (0.48)	2.92 (194)	.01
Nonadherence	1.50 (0.52)	1.90 (0.64)	5.06 (194)	.001
Self-reported health problem	1.30 (0.46)	1.75 (0.50)	1.93 (194)	.05
Affective distress	2.45 (0.72)	2.22 (0.85)	2.11 (194)	.04

Next, we present regression models for patient-reported outcomes in Tables 3 and 4. We provide both standardized (*β*) and unstandardized regression coefficients (b). As Table 3 shows, affective distress was positively predicted by gender (*β*=.13; *P*=.05) and satisfaction with medical encounters (*β*=.34; *P*<.001). Perception of strain in medical encounters was positively associated with affective distress (*β*=.20; *P*=.01) in model 2, which became nonsignificant in model 3, probably because its main effect was partially out when the interaction terms were included. Similarly, education and eHealth literacy were negative predictors of affective distress in model 2 before including the interaction terms (*β*=–.18, *P*=.03 and *β*=–.32, *P*=.01, respectively). There was also a significant interaction between education and eHealth literacy in predicting affective distress (*β*=–.60; *P*=.05), which indicated that higher levels of education predicted lower averages of feeling anxiety and worry despite lower levels of eHealth literacy among older internet users.

As shown in Table 4, age (*β*=–.17; *P*=.02), gender (*β*=–.22; *P*=.01), health-related internet use (*β*=.29; *P*=.03), and medical satisfaction (*β*=–.28; *P*=.01) were significant predictors of experiencing a health problem associated with the use of information found on the internet. There was also a significant interaction between education and perception of strain in medical encounters in predicting self-reported health problems. The association was greater among respondents with lower levels of education (*β*=–.55; *P*=.04), which indicated that the association between experiencing a health problem in relation to health-related internet use and perception of strained medical encounters was greater among respondents with lower levels of education. The regression models explained 23% of the variance in affective distress and 18% of the variance for self-reported health problems.

**Table 3:** Regression analyses predicting affective distress (N=194).

Covariates	Affective distress										
	Model 1^a^				Model 2^b^				Model 3^c^		
	b	*β*	*P* value	b		*β*	*P* value	b		*β*	*P* value
Age	–0.01	–0.07	.41	–0.01		–.07	.36	–0.01		–0.08	.23
Sex	0.13	0.08	.24	0.20		.12	.08	0.21		0.13	.05
Race	–0.01	–0.00	.91	0.03		.01	.92	0.05		0.02	.81
Education	–0.20	–0.29	<.001	–0.12		–.18	.03	0.40		0.59	.10
Income	0.16	0.21	.02	0.14		.19	.03	0.12		0.16	.08
Marital status	–0.12	–0.07	.62	–0.19		–.11	.26	–0.19		–0.11	.29
Health-related internet use				0.02		.02	.89	0.03		0.02	.88
eHealth literacy				–0.31		–.32	.01	–0.25		–0.26	.42
Medical satisfaction				0.40		.33	.001	0.41		0.34	.001
Perceived strain				0.20		.20	.01	0.11		0.11	.40
Nonadherence				0.04		.02	.77	0.03		0.02	.74
Education × strain								–0.09		–0.34	.15
eHealth literacy × strain								0.08		0.27	.43
eHealth literacy × education								–0.12		–0.60	.05

^a^*R*^2^ for model 1 was 0.09 (adjusted *R*^2^=0.06).

^b^*R*^2^ for model 2 was 0.22 (adjusted *R*^2^=0.16).

^c^*R*^2^ for model 3 was 0.23 (adjusted *R*^2^=0.17).

**Table 4:** Regression analyses predicting self-reported health problems (N=194).

Covariates	Self-reported health problem										
	Model 1^a^				Model 2^b^				Model 3^c^		
	b	*β*	*P* value	b		*β*	*P* value	b		*β*	*P* value
Age	–0.01	–0.17	.02	–0.01		–.16	.02	–0.01		–.17	.02
Sex	–0.10	–0.20	.01	–0.12		–.22	.01	–0.11		–.22	.01
Race	–0.06	0.19	.17	–0.08		–.11	.10	–0.07		–.10	.12
Education	–0.02	–0.10	.22	–0.03		–.14	.11	0.10		.48	.21
Income	–0.00	–0.01	.89	0.01		.02	.74	–0.00		–.02	.89
Marital status	–0.04	–0.06	.13	–0.02		–.03	.25	–0.01		–.01	.34
Health-related internet use				0.13		.30	.03	0.12		.29	.03
eHealth literacy				0.02		.05	.62	0.02		.02	.95
Medical satisfaction				–0.11		–.27	.01	–0.11		–.28	.01
Perceived strain				0.04		.12	.34	0.10		.31	.32
Nonadherence				0.01		.02	.70	0.01		.03	.68
Education × strain								–0.05		–.55	.04
eHealth literacy × strain								0.01		.14	.68
eHealth literacy × education								–0.01		–.15	.67

^a^*R*^2^ for model 1 was 0.08 (adjusted *R*^2^=0.05).

^b^*R*^2^ for model 2 was 0.16 (adjusted *R*^2^=0.11).

^c^*R*^2^ for model 3 was 0.18 (adjusted *R*^2^=0.11).

## Discussion

### Principal Findings

In this study, we provide the empirical evidence of the importance of education and eHealth literacy and their implications for health-related outcomes within the context of transactional importance of medical encounters in older demographics. Our findings highlight the role of education as a significant moderator of the effects of inadequate eHealth literacy and strained medical encounters on patient-reported outcomes. Specifically, older internet users with lower levels of eHealth literacy but higher educational levels reported feeling less worried and anxious because of what they read on the internet. For example, the significant interaction between educational level and perceived strain in medical encounters suggested that the effect of lower education on likelihood of experiencing a health problem, associated with information use obtained from the internet, is greater under conditions of greater strain in medical encounters.

Gender is a significant predictor of patient-reported outcomes. Older women reported lower averages on experiencing a health problem but higher averages on affective distress because of using the information obtained from online sources. These associations could be attributed to their gender-associated caretaker roles and responsibilities that encourage women to be more discerning health information consumers while increasing their exposure to potentially inaccurate information that may increase their distress level as women tend to be more frequent users of the internet for health-related information [16,65-68]. In contrast, older men reported higher averages on experiencing a health problem as consequence of using the internet information but less affective distress. These different gender-based outcomes need further exploration to have a more comprehensive grasp of the nature of the effect of health information–related use of the internet on subjective health outcomes.

Nonadherence with medical professionals was not a significant predictor of patient-reported outcomes. As Seckin et al [51] noted, prior to the 1980s, the passive patient was expected to accede to their physician’s authority by conforming to their physician’s stipulated treatment and advice. Socialization of older cohorts into medical paternalism, which promoted a *doctor knows best* approach for health care increased the tendency of older adults to show compliance with medical professional authority, which offers a potential explanation for this specific nonsignificant association in the older sample [1,26].

Satisfaction with medical encounters is a negative predictor of likelihood of experiencing a health problem associated with internet information. Respondents who were more satisfied with their patient-physician relationship may feel less need to consult online sources of information, which may or may not be credible, thereby lowering their risks with incorrect information or misinterpretation of correct information. Alternatively, even if they consult the internet for supplementary information, they might be less likely to implement the information or follow the advice found online because of their trust in their health care provider’s approach to their care provision. Interestingly, satisfaction with medical encounters is positively associated with affective distress, which might reflect increased information or attention received from health care providers when a patient feels distressed because of the content of the information they came across on the internet. It is also important to note that perception of strain in doctor-patient relationships was a significant predictor of affective distress. Perception of strain in medical interactions, as discussed earlier in the paper, may discourage people from discussing online information with their health care providers, which in turn may increase their distress level, particularly when a patient does not possess adequate health literacy skills to evaluate the information [32].

### Limitations

This paper captures limited dimensions of patient-reported outcomes. Individuals with chronic health issues or serious diseases may use the internet in more targeted ways than those who browse the internet for general health purposes, which in turn may result in differential health outcomes and perceptions of how medical encounters are affected by use of the internet sources. Future evaluations of health-related internet use should focus on older adults with specific chronic conditions to elucidate its role in health management. Furthermore, the analyses relied on self-reports and reflected on the cross-sectional nature of these associations. A longitudinal design to elucidate the pathways through which health-related internet use influences health outcomes will provide more detailed information [21,69,70]. Thus, future work should consider the specific mechanisms such as behavioral pathways (eg, specific self-care behavior) that potentially link eHealth information consumerism to health outcomes. Using a mixed-methods approach will also help to unpack health providers and consumers’ perspectives. Inclusion of unaccounted variables, such as trust in health care providers or trust in the internet, would have probably increased the explanatory power of the statistical models used in this study.

Despite these limitations, this study makes an important contribution to research on health-related internet use among older adults by illustrating the empirical links of education and eHealth literacy to patient-reported outcomes [25,71]. There has been a research lag in examining whether, to what extent, and how eHealth literacy influences patient-reported outcomes in the general population, particularly among older adults [9,23,34,69]. This paper captures the role of eHealth literacy among older internet users. The results highlight the need to foster positive experiences in medical interactions and underlie the importance of informed consumerism of online information among older adults in the age of eHealth information technology.

### Conclusion

The findings have implications for health care providers to guide patients to reliable and accurate health resources on the internet. Older health consumers will be able to make more informed choices and better decisions about their health if health professionals help to empower them in finding credible and trustworthy online sources [9,33,34,43,47,48,69,72]. Given older adults’ substantial health needs, their ability to find credible online information is critical in furthering a research agenda on technology use among older adults [2,23,73-75]. Empowerment of older adults as proactive health information consumers necessitates addressing their eHealth literacy needs and improving their health literacy skills through educational or intervention programs, which in turn will help to offset potential undesirable outcomes due to misinformation or inaccurate information use [1,14,24,36,76].

## Appendix

Multimedia Appendix 1 Measures.

## Abbreviations

e-HLS: eHealth Literacy Scale
KN: Knowledge Networks
TMC: Transactional Model of Communication
TMeHL: Transaction Model of eHealth Literacy
