# Supplementary material for: Patient-Reported Outcomes in a Nationally Representative Sample of Older Internet Users: Cross-sectional Survey
Source: JMIR Aging. 2021 Nov 24;4(4):e16006. doi: 10.2196/16006 (PMC8663692; doi:10.2196/16006)
Supplement: Multimedia Appendix 1 [file aging_v4i4e16006_app1.docx]

**Multimedia Appendix 1: Measures**

*Health-related Internet use*

1. Do you seek information on the Internet to self-diagnose or find a diagnosis for other people?

1. Never

2. Rarely

3. Sometimes

4. Most of the time

5. Always

2. Do you use information from the Internet to identify appropriate treatments for yourself?

1. Never

2. Rarely

3. Sometimes

4. Most of the time

5. Always

3. Do you request medications from a health care provider based on the Internet information?

1. Never

2. Rarely

3. Sometimes

4. Most of the time

5. Always

4. Do you request medical examinations from a health care provider based on the Internet

information?

1. Never

2. Rarely

3. Sometimes

4. Most of the time

5. Always

5. Do you request treatments from a health care provider based on the Internet information?

1. Never

2. Rarely

3. Sometimes

4. Most of the time

5. Always

6. Do purchase medications based on the Internet information without necessarily talking to a

doctor?

1. Never

2. Rarely

3. Sometimes

4. Most of the time

5. Always

7. Do you treat a health issue based on Internet information?

1. Never

2. Rarely

3. Sometimes

4. Most of the time

5. Always

8. Do you use information from the Internet to make treatment decisions?

1. Never

2. Rarely

3. Sometimes

4. Most of the time

5. Always

9. Information on the Internet helps me to communicate more effectively with a health care

provider during appointments.

1. Strongly disagree

2. Disagree

3. Somewhat agree

4. Agree

5. Strongly agree

10. Information on the Internet helps me to ask more informed questions to health providers.

1. Strongly disagree

2. Disagree

3. Somewhat agree

4. Agree

5. Strongly agree

11. Information on the Internet helps me to better understand what my health provider is telling

me during appointments.

1. Strongly disagree

2. Disagree

3. Somewhat agree

4. Agree

5. Strongly agree

12. I receive more attention to my questions from health providers as a result of gathering health

or medical information from the Internet.

1. Strongly disagree

2. Disagree

3. Somewhat agree

4. Agree

5. Strongly agree

13. I receive more information from health care providers as a result of gathering health or

medical information from the Internet.

1. Strongly disagree

2. Disagree

3. Somewhat agree

4. Agree

5. Strongly agree

14. Interactions of health care providers with me have become more respectful as a result of

gathering health or medical information from the Internet.

1. Strongly disagree

2. Disagree

3. Somewhat agree

4. Agree

5. Strongly agree

*e-Health Literacy*

15. Do you read disclosure statements on health websites?

1. Never

2. Rarely

3. Sometimes

4. Most of the time

5. Always

16. Do you check for credentials and institutional affiliations of those who provide information

on health websites?

1. Never

2. Rarely

3. Sometimes

4. Most of the time

5. Always

17. Do you check a website’s sponsor(s)?

1. Never

2. Rarely

3. Sometimes

4. Most of the time

5. Always

18. Do you the check ownership of a health website?

1. Never

2. Rarely

3. Sometimes

4. Most of the time

5. Always

19. Do you check for financial ties between website information and the website’s sponsor(s)?

1. Never

2. Rarely

3. Sometimes

4. Most of the time

5. Always

20. Do you appraise the adequacy and integrity of information providers’ credentials?

1. Never

2. Rarely

3. Sometimes

4. Most of the time

5. Always

21. Do you check to see whether a physical address is provided?

1. Never

2. Rarely

3. Sometimes

4. Most of the time

5. Always

22. Do you check for stated goals and objectives?

1. Never

2. Rarely

3. Sometimes

4. Most of the time

5. Always

23. Do you appraise whether coverage of health topics is clear and comprehensive?

1. Never

2. Rarely

3. Sometimes

4. Most of the time

5. Always

24. Do you check whether other print or web resources confirm information provided?

1. Never

2. Rarely

3. Sometimes

4. Most of the time

5. Always

25. Do you check whether information is current and updated?

1. Never

2. Rarely

3. Sometimes

4. Most of the time

5. Always

26. Do you check the last time the information was updated?

1. Never

2. Rarely

3. Sometimes

4. Most of the time

5. Always

27. How often do you ask health professionals for advice about where to find credible

information on the Internet?

1. Never

2. Rarely

3. Sometimes

4. Most of the time

5. Always

28. I trust the Internet for obtaining accurate health information?

1. Strongly disagree

2. Disagree

3. Somewhat agree

4. Agree

5. Strongly agree

29. I believe information provided on the Internet is credible.

1. Strongly disagree

2. Disagree

3. Somewhat agree

4. Agree

5. Strongly agree

30. I believe the information provided on the Internet is balanced and accurate.

1. Strongly disagree

2. Disagree

3. Neither agree nor disagree

4. Agree

5. Strongly agree

31. I think the information provided on the Internet is the same as or better than what most health

professionals provide.

1. Strongly disagree

2. Disagree

3. Somewhat agree

4. Agree

5. Strongly agree
